# Real-World Evidence of 3D Printing of Personalised Paediatric Medicines and Evaluating Its Potential in Children with Cancer: A Scoping Review

**DOI:** 10.3390/pharmaceutics16091212

**Published:** 2024-09-14

**Authors:** Munsur Ahmed, Stephen Tomlin, Catherine Tuleu, Sara Garfield

**Affiliations:** 1 Pharmacy Department, Great Ormond Street Hospital for Children NHS Foundation Trust, London WC1N 3JH, UK; munsur.ahmed@gosh.nhs.uk; 2 School of Pharmacy, University College London, London WC1N 1AX, UK; s.garfield@ucl.ac.uk; 3 Children’s Medicine Research & Innovation Centre, Great Ormond Street Hospital for Children NHS Foundation Trust, London WC1N 3JH, UK; stephen.tomlin@gosh.nhs.uk

**Keywords:** 3D printing, personalised medicine, paediatric oncology, medication acceptability, scoping review

## Abstract

Personalised medicine, facilitated by advancements like 3D printing, may offer promise in oncology. This scoping review aims to explore the applicability of 3D printing for personalised pharmaceutical dosage forms in paediatric cancer care, focusing on treatment outcomes and patient experiences. Following the Joanna Briggs Institute (JBI) methodology, a comprehensive search strategy was implemented to identify the relevant literature across databases including PubMed, Embase, and Web of Science. Three independent reviewers conducted study selection and data extraction, focusing on studies involving paediatric patients under 18 years old and pharmaceutical dosage forms manufactured using 3D printing technology. From 2752 records screened, only six studies met the inclusion criteria, none of which specifically targeted paediatric cancer patients. These studies examined aspects of acceptability, including swallowability, taste, and feasibility of 3D-printed formulations for children. While the studies demonstrated the potential benefits of 3D printing in paediatric medication, particularly in personalised dosing, there is a notable lack of evidence addressing its acceptability in paediatric cancer patients. Further interdisciplinary collaborative research is needed in this area to fully assess preferences and acceptability among children with cancer and their parents or caregivers.

## 1. Introduction

In recent years, 3D printing technology has emerged as a promising tool in the field of pharmaceuticals, offering the potential to revolutionize medication manufacturing and administration [1,2], particularly in paediatric populations [3,4,5,6,7]. This innovative approach enables the creation of personalised dosage forms tailored to individual young patient needs, such as responsive fast adaptation at ‘bedside’ of:

- Dose (low, flexible, or precise) [8,9],
- Potential for drug combination [10,11,12,13,14,15],
- Drug release profiles (from fast to immediate and further modified release) [10,11,12,13,16],
- Drug product/dosage form types (size, shape, colour, flavour, texture) [17,18,19,20,21,22,23,24,25].

In turn, it presents unique opportunities for improving medication adherence, safety, and therapeutic outcomes. In the realm of paediatric oncology, where treatment adherence is critical for achieving favourable outcomes, the application and implementation of 3D printing technology may hold significant promise.

Despite the growing interest and potential benefits of 3D-printed medications, especially around technical aspects and the feasibility of 3D printing of pharmaceuticals [26,27,28,29,30,31,32,33], a literature review by Li et al. [34] shows a lack of published literature on 3D-printed oral solid dosage forms for cancer applications in adults. They identified a single study where researchers printed an anti-cancer drug 5-fluorouracil using 3D printing [35] and performed in vitro dissolution studies. To date, there is no FDA-approved anti-cancer 3D-printed oral drug on the market.

By advancing our understanding of the role of 3D-printed medications in paediatric cancer care, we can ultimately improve treatment outcomes for children facing this challenging disease Therefore, providing a more targeted investigation into the acceptability of 3D-printed medications among paediatric patients with an additional focus on paediatric cancer patients not only could address a critical gap in the literature but also identify areas for further research and development.

This scoping review searched for evidence relating to the application of the 3D printing of drug formulations in children and the benefits it could bring to this very specific cancer patient population. Unlike earlier scoping reviews by Abeysekera et al. [36], Cooke et al. [37], Ribeiro et al. [38], or Pradíes et al. [39], all of which primarily focused on 3D printing of anatomical/surgical models, implants or devices, and Ianno et al. [40], which focused on printing technologies, or by Lafeber et al. [41], which primarily focused on the feasibility and potential advantages of 3D printing technology in paediatric medications, the present review specifically explores the real-world evidence of the acceptability of 3D-printed pharmaceutical dosage forms in children, with a particular emphasis on its application in paediatric cancer. More recent literature is also searched for in this rapidly progressing area.

By advancing our understanding of the role of 3D-printed medications in paediatric cancer care, we can ultimately improve treatment outcomes for children facing this challenging disease.

## 2. Review Question

What is the real-world evidence of the acceptability of 3D printing dosage forms and its application in children and, specifically, in paediatric cancer?

## 3. Methods

### 3.1. Inclusion Criteria

- Population

Children under 18 years old, including neonates and infants were included in this study. Any articles aimed at the adult population or on adult healthy subjects were excluded.

- Concept

Studies involving manufacturing pharmaceutical dosage forms for human use with 3D printing techniques were included. 

Human and non-human in vitro studies, 3D printing techniques and optimisations, 3D-printed dosage optimisations, 3D printed medical devices, usage in regenerative medicine, 3D printing in imaging, scaffolding, surgical implants, and organs/tissue modelling studies were excluded.

- Context

This review was conducted in two separate contexts: 

Guided by our preliminary search where we predicted to find a lack of reports on paediatric cancer, we performed our search criteria in two distinct parts. First, we searched sources for studies with cancer being the context. Then we searched the literature without cancer being the context.

Thus, we aimed to scope the literature for acceptability studies of 3D-printed formulations carried out in children in general and with the additional context of any cancer type. 

An overview of the inclusion and exclusion criteria is provided in Table 1.

### 3.2. Types of Sources

Following the Joanna Briggs Institute (JBI) template for scoping reviews, this scoping review considered all accepted study designs, such as randomized controlled trials, non-randomized controlled trials, individual case reports, case–control studies, cross-sectional studies, case series, prospective and retrospective cohort studies, descriptive cross-sectional studies, and qualitative studies.

Any studies that did not collect primary data were excluded. All reviews, in vitro studies, non-human or animal studies, 3D printing as scaffolding, use in surgical procedures, usage in imaging, and tissue or organ modelling were also excluded.

This review followed the JBI methodology for scoping reviews [42], utilizing the population/concept/context (PCC) framework.

### 3.3. Search Strategy

The search strategy was designed to locate both published and unpublished research. Initially, a preliminary search was conducted on MEDLINE (PubMed) to find relevant studies. The information within the titles and abstracts of these papers, along with the index terms used to categorize them, helped in developing a comprehensive search strategy for MEDLINE (PubMed). This strategy, which included all relevant identified index terms and keywords, was then tailored to each database and information source used (see Appendix A for the Embase search strategy). Additionally, the reference lists of all included sources were reviewed to find further relevant studies.

Studies published in the English language were included. No restrictions were made regarding the year of publication or the design of the study.

The databases searched included PubMed, Embase, Web of Science, CINAHL databases, Cochrane database, Emcare, Scopus, International Pharmaceutical abstracts (IPA), Prospero, Episteminokos, and Clinicaltrials.gov. Sources of unpublished studies and grey literature searched included Google Scholar.

### 3.4. Search Process 

Rayyan [43], a web and mobile app for systematic reviews, was used to collate and analyse all identified uploaded citations. Duplicate entries were removed. Blinded by Rayyan, three independent reviewers screened the titles and abstracts to determine their eligibility based on the inclusion criteria (two of the reviewers screened 10% of the records and the third 100%). Full texts of potentially relevant sources were retrieved and imported into Rayyan. The full text of the selected 10% of citations was thoroughly and independently reviewed against the inclusion criteria by all three reviewers. All disagreements between the reviewers noted during the selection process were resolved through discussion. Cohen’s Kappa, k, was calculated for interrater reliability. Between reviewers CT and MA, k was 1, indicating perfect agreement. Between reviewers SG and MA, k was 0.38 indicating a fair agreement. It was thus not deemed necessary for the two reviewers to screen the remaining 90% of the papers.

### 3.5. Data Extraction

Data were extracted from papers included in the scoping review following the PCC framework through Rayyan. Included articles were screened on the characteristics of population, concept, and context. Data were extracted relating to the acceptance, feasibility, and preference of children for 3D-printed tablets and clinical applications. The full texts of the included articles were independently reviewed by all three reviewers and the rate of agreement was 100%. 

### 3.6. Quality Assessment

To assess and compare the included studies with diverse study designs uniformly, the tool developed by Sirriyeh et al. [44] (Quality Assessment Tool for Studies with Diverse Designs (QATSDD)) was used to appraise the studies. The face validity and inter-rater reliability of this tool has previously been established [45]. All included studies were appraised and included for data synthesis.

### 3.7. Data Synthesis

Thematic synthesis was used to synthesize data for new findings by following a three-step process: line-by-line coding of the primary texts (step 1) to construct descriptive themes (step 2), followed by generation of analytical themes (step 3) using ‘third order’ interpretations of the descriptive themes [45].

## 4. Results

### 4.1. Study Inclusion

In searches inclusive of neoplasm or cancer as a context, a total of 225 records were identified, 12 of which were removed as duplicates. All the identified records (213) were excluded as they did not meet the inclusion criteria. As a result of there being no specific records in cancer as a context, searches without neoplasm as a context was taken forward.

In searches without neoplasm or cancer as a context, a total of 2752 records were identified in our search from all the databases and resources mentioned. Of these records, 339 records were excluded as duplicates. 

Out of the remaining 2413 records, titles and abstracts were screened and 2400 records were excluded as these did not meet the inclusion criteria.

**Figure 1:**
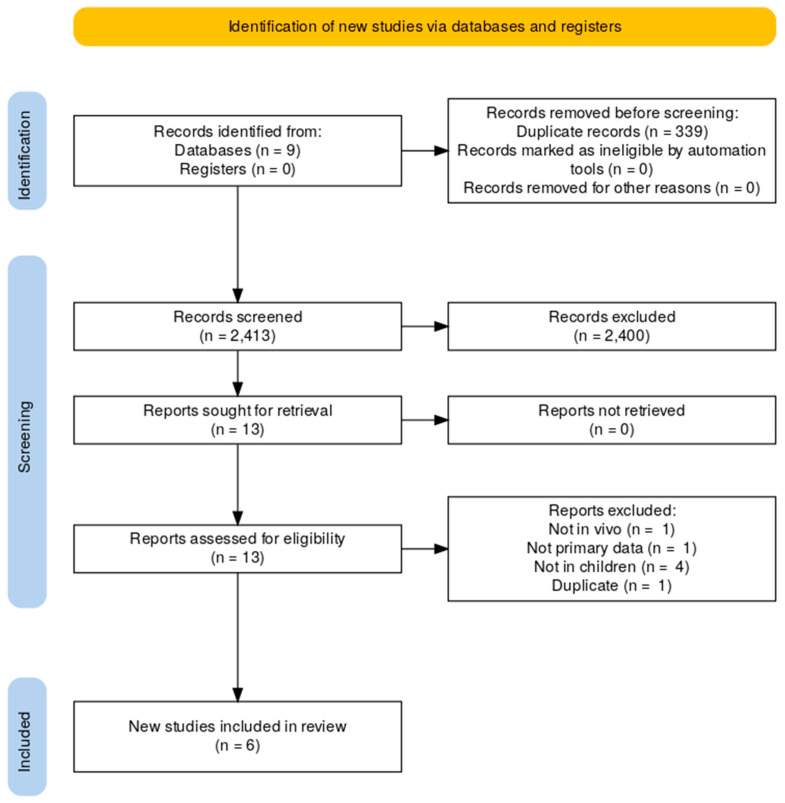
Flow chart of inclusion of reports without neoplasm as context.

The flow chart of the inclusion of reports is shown in Figure 1. The eligible records excluded during the screening process are given in Appendix B.

### 4.2. Characteristics of Included Studies

Five of the six studies were observational, including focus groups and questionnaires, and one was an experimental study. Additional characteristics of the included studies are provided in Table 2. 

### 4.3. Quality of Included Papers

The QATSDD scores of the included studies varied from 10% to 81% and are provided in Table 3 below. 

Studies varied in their quality and limitations. Some studies had limitations in generalisability, such as small sample sizes [46,50,51], single-centre studies [32,34,35], a small age range of participants [49,51], or limited scope (e.g., placebo administration in healthy children) [47]. Others had limited outcomes and did not assess factors such as the size and shape of the 3D-printed tablets [47], colour [47], texture, or comprehensive flavour preferences [46,47]. Others lacked cost analysis to assess the cost-effectiveness of 3D printing over conventional methods [49,50,51], long-term follow-ups [33], long-term stability data for information on the shelf-life of 3D-printed drugs [50,51], and/or lack of assessment of long-term acceptability or usability of 3D-printed formulations [46,47]. 

Limitations in study design included the absence of control groups [46,47,50] and lack of blinding, which may introduce bias in clinical outcome evaluations [46,49]. One study lacked patient perspectives [48]. 

### 4.4. Synthesis of Results

#### Thematic Analysis

Seven main themes emerged from the included studies: 

1. Clinical applications, 2. Acceptability of 3D-printed dosage forms in the paediatric population, 3. Benefits of 3D-printed drug products, 4. Concerns regarding the implementation of 3D printing of drug products, 5. Feasibility of 3D-printed dosage forms, 6. Suggestions for printed medicines, and 7. Potential applications and implications.

1. Clinical application:

Three studies [49,50,51] were identified that applied 3D-printed medicines to children in clinical settings. The studies assessed the clinical efficacy and safety of 3D-printed dosage forms, including their ability to maintain therapeutic levels, control blood parameters, and manage specific medical conditions such as maple syrup urine disease (MSUD).

Collectively, the findings from the three studies suggests that 3D-printed drugs can be implemented in clinical practice. Two studies [49,50] used measurements of blood levels to assess the ability of 3D-printed formulations to maintain blood levels within the target range compared to conventional dosage forms prepared by manual compounding. One assessed this in patients with maple syrup urine disease (MSUD) and the other with patients with transient hypothyroxinaemia of prematurity. Evidence from these two studies [49,50] suggests that 3D printing technology is a superior method for adapting drug dose accurately and can improve quality and drug efficacy. In MSUD, they found that both types of formulations effectively regulated isoleucine blood levels in patients; however, the mean levels of isoleucine with the 3D formulation were found to be more closely aligned with the target value and exhibited reduced variability compared to the standard capsules [50]. In transient hypothyroxinaemia of prematurity, the quality of manually subdivided drugs failed to meet industry standards, whereas the 3D-printed drugs did. The thyroid hormone levels of infants treated with 3D-printed levothyroxine sodium tablets showed significant elevation compared to the manual groups. Additionally, the 3D-printed tablets showed advantages in terms of faster initiation of suckling, fewer days of intravenous nutrition, and higher cumulative weight gain in preterm infants [49]. A third study [51] did not provide details of any assessment after administration; however, the 3D-printed drugs conformed with pharmacopeial standards and were reported as being acceptable to family members and healthcare professionals in clinical settings.

2. Acceptability of 3D-printed dosage forms in paediatric population:

The 3D-printed oral dosage forms were found to be favourable by patients [46,47,50] and healthcare professionals [48,51] compared to conventional dosage forms.

Studies examined the factors influencing the acceptability of 3D-printed dosage forms among paediatric patients, including visual preference, swallowability, mouthfeel, flavour, and colour.

Data from a well-designed study with a large sample size provides strong support for the preferences of children regarding 3D-printed tablets based on visual preference [47] (appearance, texture, perceived taste, and familiarity) and indicates no significant difference in the impact of age and sex. Chewable formulations of 3D-printed drugs were preferred by children [47] over oro-dispersible formulations or from swallowing the tablet whole.

The size of the tablets was found to be important for swallowability, with larger tablets found to be difficult to swallow [46]. Evidence relating to preferred colours and flavours was limited to four patients and hence no meaningful conclusions could be drawn [50].

Healthcare professionals considered 3D-printed tablets to have accurate doses, good appearance, and improved patient adherence and medication safety [48,51].

3. Benefits of 3D-printed drug products:
  All included studies highlighted the perceived benefits of 3D-printed drug products over conventional formulations. These included precise and personalised doses [47,48,50,51], polypills [48], improved drug acceptance [46,47,48,50,51], reduced labour intensity [51], cost savings [48,51], improved safety [49,50,51], and rapid manufacture [47,50].
  - Precise and personalised doses: Healthcare professionals highlighted the advantage of 3D printing technology in producing precise and patient-specific doses, enhancing medication adherence and treatment outcomes [48]. Healthcare professionals also considered that it was useful to have an option of on-demand new and modified doses based on the patient’s new requirements [47]. Preliminary studies focusing on the feasibility of producing 3D-printed tablets have found that 3D printing has the potential to overcome challenges in pharmaceutical manufacturing, such as the ability to produce complex geometries and personalised dosage forms [50]. The benefits of 3D printing over conventional manufacturing [35] were found to be accurate dosing and compliance with the specifications of the regional Pharmacopoeia [51]. The 3D printing technology allowed for precise control over the dose of the subdivided tablets [51]. Moreover, the model parameters of the 3D printer were able to be adjusted to obtain tablets with any desired dose, ensuring accurate dosing for individual patients [51].
  - Polypills: Healthcare professionals were of the view that incorporating multiple drug substances into one product simplifies medication regimens, particularly for children with polypharmacy, thereby improving adherence [48].
  - Improved drug acceptance: Children [47,50] and healthcare professionals [48,50] were of the view that customizing the shape, size, and colour of solid dosage forms can enhance drug acceptability, especially among paediatric patients. Evidence for the preference for shape and colour is limited. Swallowability, palatability, such as mouthfeel, and taste positively influenced acceptability in a sample of children [46]. Suggestions from healthcare professionals included using funny or appealing shapes to make medication more appealing to children [48]. Accurate doses and good appearance were considered by nurses and pharmacists to contribute to improving drug acceptability [51].
  - Reduced labour intensity: 3D printing is considered by healthcare professionals to have benefits over the traditional methods of manipulating dosage forms by hand, such as splitting, crushing, powdering, or making an extemporaneous liquid preparation—all require manual labour and can be time consuming for healthcare professionals and caregivers [51]. Healthcare professionals were of the view that 3D printing could potentially improve efficiency by reducing the workload through the automation process [51].
  - Cost savings: In the view of healthcare professionals, automation will eliminate the need for additional equipment such as tablet cutters and crushers, extemporaneous liquid formulations, and reduce the costs of human resources and materials [51]. Healthcare professionals, especially pharmacists, were of the view that 3D printing technology could potentially reduce the costs associated with making tablets by reducing waste costs [48]. Nevertheless, significant upfront costs might be necessary for advanced 3D printers, as well as ongoing maintenance expenses and expenditures for materials, equipment, and personnel, could result in manufacturing being costly [48]. As the evidence is still limited, further studies are required to demonstrate the cost effectiveness of implementing 3D printing technology in hospital settings.
  - Improved safety: Results from the preliminary studies indicate that 3D printing technology will ensure the accuracy and consistency of dosages with drug uniformity [51]. The laboratory tests performed showed exceptionally low variations in mass, drug content, and uniformity compared to manual splitting, reducing the risk of dosage errors from subdividing doses [49,51]. Improved safety results were also obtained from the quality control and monitoring of 3D-printed tablets [51] and the elimination of cross-contamination [49,50]. Personalised dosing will contribute to improved safety by reducing the side effects from inappropriate doses.
  - Rapid manufacture: 3D printing allows for on-demand production of drug dosage forms at the point of dispensing with a small-batch and rapid manufacturing process. This can improve access to medicines and reduce lead times, especially for children with specific needs [47]. It is fast and more efficient, reducing the time and resources needed for making tablets compared to conventional manufacturing [50]. Twenty-eight 3D-printed tablets, sufficient for one month of treatment, were printed in approximately 8 min [50].
4. Concerns regarding the implementation of 3D printing of drug products [48]:
  Concerns were raised by healthcare professionals surrounding the implementation of 3D-printed pharmaceutical dosage forms [48]. The main concerns were:
  - Medication safety: Healthcare professionals (physicians, nurses, pharmacists) expressed concerns about the even distribution of drug substances within printed dosage forms (verification of drug contents), accuracy of doses, quality control, stability, and shelf-life and storage conditions of formulations [48].
  - Drug administration: Healthcare professionals also expressed their concerns on drug administration. Challenges include administering dosage forms to all paediatric patients, especially infants and those with enteral feeding tubes, as well as concerns about functionality and the dissolution characteristics of patient-specific dosage forms [48].
  - Production and delivery on demand: The logistics and response time for the production and delivery of on-demand prepared dosage forms (the ability to react quickly to dose changes) were noted concerns by healthcare professionals, particularly in hospital settings [48].
  - Cost: Concerns were raised on the cost effectiveness of personalised drug products by healthcare professionals [48]
5. Feasibility of 3D-printed dosage forms:

Three-dimensional printing of personalised dosage forms has been evaluated for quality and feasibility [46,49,50,51] and found to have enhanced drug delivery profiles [49], excellent content uniformity and stability [51], conformance and compliance with pharmacopeial standards [51], good accuracy and reproducibility [46], acceptable mechanical properties, and acceptable dissolution profiles [50], indicating their suitability for diverse formulations.

6. Suggestions for printed medicines

Healthcare professionals suggested various drug substances and medical conditions where 3D-printed pharmaceutical drug products would be advantageous, including personalised doses, oral dosage forms, controlled release formulations, oro-dispersible dosage forms, and combination products for various medical conditions such as tuberculosis, HIV, organ transplantation, and cancer [48]. They suggested the need for interdisciplinary collaboration, patient involvement in the design process, and integration of 3D printing into existing healthcare systems.

7. Potential applications and implications:

With all the benefits listed above, 3D printing can increase the potential for innovations in drug formulations and developments. Preliminary studies focusing on the feasibility of producing 3D-printed tablets allow potential usages in combination with other drugs or biologics, or as drug-loaded medical devices, for on-demand production of orphan drugs [50], immediate and controlled release formulations, and implants [51].

Although the aforementioned studies collectively offer valuable insights into acceptability and personalised dosing with 3D printing technology, none specifically targeted paediatric cancer patients. Nonetheless, the findings underscore the potential of 3D printing in enhancing medication acceptability and the clinical outcomes in paediatric oncology, emphasizing the need for further research in this critical area.

## 5. Discussion

The scoping review included six studies focusing on the acceptability and clinical application of 3D printing technology in children, two of which have been published since the previous review by Lafeber et al. [41]. The studies varied in design, including observational studies, questionnaire surveys, focus group discussions, and prospective crossover experimental studies.

The thematic synthesis identified several potential advantages associated with 3D-printed drug products compared with conventional forms, showcasing their potential to revolutionize medication delivery. Notable benefits include heightened precision in dosing, the ability to tailor formulations to individual patient needs, and the potential for improved patient adherence and therapeutic outcomes.

This review has explored the acceptability of 3D-printed dosage forms among paediatric populations, recognizing the unique considerations and requirements of this demographic. Healso evaluated the quality and feasibility of 3D-printed formulations, providing insights into the technical intricacies and practical considerations associated with their production and administration.

Ultimately, the review underscores the transformative potential of 3D printing in pharmaceutical manufacturing, envisioning a future where personalised medicine and on-demand manufacturing are seamlessly integrated into clinical practice.

Our review builds on the previous review by Lafeber et al. [41], with two additional studies published [46,49] thenceforth. We also searched for published evidence of 3D printing in paediatric oncology. Our review confirms their findings and provides additional data for the acceptability and clinical applications of 3D-printed tablets. These include 3D printing as a superior method for adapting drug doses accurately using blood level assessments [49], favourability by patients [46], improved drug acceptance [46], improved safety [49], swallowability and palatability positively influencing acceptability [46], reduced dosing errors [49], elimination of contamination [49], enhanced delivery profiles [49], good accuracy and reproducibility [46], and others. A recent study also confirms the benefits of combining two actives in the same formulation and patient acceptability of chewable formulations [52]. We have also identified the absence of studies in paediatric cancer.

While the existing evidence provides valuable insights into the acceptability of 3D-printed medications in paediatric populations, it is essential to recognize and acknowledge the limitations of the included studies. Notably, the absence of studies focusing specifically on paediatric cancer patients limits our understanding of their unique preferences and challenges regarding medication acceptability in order to leverage 3D printing capabilities for this patient population. Moreover, the small sample sizes and limited scope of some studies underscore the need for larger-scale research initiatives to validate and generalize the findings.

Our review used thematic analysis where some conceptually poor or ‘thin’ data contributed less to the overall analysis compared to studies with ‘thick’ or higher-level conceptual data. The studies varied greatly in quality too. Nevertheless, there was a consistency of themes across studies suggesting cumulative validity of findings. Our review was also limited to the English language and may have resulted in excluding some useful studies.

Despite the promising prospects, the analysis uncovered a series of challenges that might hinder the widespread adoption of 3D printing in pharmaceutical manufacturing. Regulatory complexities pose challenging barriers requiring meticulous navigation to ensure compliance and safety standards. Quality assurance emerges as a paramount concern, necessitating rigorous protocols to guarantee the reliability and consistency of 3D-printed medications. Additionally, there is a need for innovative approaches to address production scalability and cost effectiveness.

Successful integration of 3D printing technologies into hospital settings demands a multifaceted approach. Establishing robust regulatory frameworks is imperative to provide a clear pathway for approval and deployment. Infrastructure development is essential to support the technical requirements of 3D printing facilities, while comprehensive workforce training programs are indispensable to equip personnel with the necessary skills and knowledge.

None of the included studies specifically targeted paediatric cancer patients, highlighting a critical research gap in the literature about the application of 3D printing in this population. Notwithstanding the lack of direct evidence in the context of paediatric cancer, the synthesized findings suggest several potential benefits of 3D-printed medications for children with cancer. For instance, some studies [46,47] demonstrate the importance of considering patient preferences, such as tablet size and appearance, in enhancing medication acceptability among children. Furthermore, other studies [50] highlight the feasibility and efficacy of 3D printing technology in preparing personalised therapies for rare metabolic disorders, showcasing the potential for tailored dosages, flavours, and colours to increase patient acceptability and adherence in other disease states. One study [49] identified, among other disease areas, cancer as one domain where 3D-printed personalised dosage forms would be of benefit to patients.

Despite these limitations, the implications of this review are twofold. Firstly, the findings underscore the importance of patient-centred approaches in medication development, particularly in paediatrics, where treatment adherence is crucial for improving outcomes. Secondly, the identified gaps in the research highlight the need for further investigation into the acceptability of 3D-printed medications among paediatric cancer patients and their caregivers, where getting the precise dose in a child-friendly formulation could contribute to chemotherapy optimisation, i.e., improved patient adherence, reduced wastage of medicines, improved medicine safety, and improved therapeutic outcome.

Moving forward, future research endeavours should prioritize addressing these gaps by conducting studies specifically focused on paediatric cancer populations. Additionally, interdisciplinary collaborations between clinicians, researchers, and technology experts are essential for advancing the development and implementation of 3D printing technology in paediatric oncology. By leveraging innovative approaches and technologies, such as 3D printing, we can strive to optimise medication management and improve the quality of care for children battling cancer and other serious illnesses.

## 6. Conclusions

In conclusion, this scoping review emphasizes the evident lack of research, specifically addressing the potential applicability and acceptability of 3D-printed formulations in paediatric cancer patients. Despite the absence of direct evidence in this context, the synthesized findings suggest a promising potential for 3D printing to enhance medication administration and adherence through personalised therapies tailored to this demographic. However, there appears to be an over emphasis on promises of 3D printing versus actual short- to mid-term clinical implementation that is driven by real, pragmatic clinical needs.

Further research is also imperative to grasp the unique challenges and preferences of children with cancer and their caregivers. This will facilitate the effective utilization of 3D-printed medications and ensure their seamless integration into clinical practice, ultimately aiming to optimise outcomes and enhance the quality of care for children undergoing cancer treatment.

## Figures and Tables

**Table 1:** Inclusion and exclusion criteria for search strategy.

Framework Element	Inclusion Criteria	Exclusion Criteria
Population	Children ≤ 18 years old	Adults (>18 years old) All non-human subjects
Concept	Manufacturing of pharmaceutical dosage forms using 3D printer	Medical devices, regenerative medicine, 3D printing in imaging, scaffolding, surgical implants, organs/tissue modelling.
Context	Neoplasm—all types	All other disease types

**Table 2:** Characteristics of included studies.

Study	Year of Publication	Active Drug	Population and Sample	Outcome	Study Design
Bracken et al. [46]	2022	placebo	30 participants (aged 4–12 years), disease not specified and included healthy volunteers.	Acceptability (swallowability, acceptability, mouthfeel, volume of water consumed, and taste)	Observational and Questionnaire
Januskaite et al. [47]	2020	placebo	368 participants (4–11 years old) school children	Acceptability (visual preference)	Questionnaire
Rautamo et al. [48]	2020	n/a	19 participants, health care professionals	Benefits, concerns, and risks of 3D; 3D drug of choice and disease area	Focus Group Discussions
Liu et al. [49]	2023	levothyroxine sodium	91 preterm infants with transient hypothyroxinaemia	Personalised dosing	Observational Study
Goyanes et al. [50]	2019	isoleucine	4 patients (3–16 years); maple syrup urine disease (MSUD)	Acceptability, flavours/colours	Prospective Crossover Experimental Study
Zheng et al. [51]	2020	spironolactone	11 hospital inpatients (<1 months–9 months)	Acceptability	Observational Study

**Table 3:** Quality assessment of all included studies following the Quality Assessment Tool for Studies with Diverse Designs (QATSDD).

QATSDD Criteria *	1	2	3	4	5	6	7	8	9	10	11	12	13	14	15	16	Total Score	% Score
Bracken et al. [46]	3	3	3	1	2	3	3	2	0	3	3	3	3	n/a	3	2	37/48	77
Januskaite et al. [47]	0	3	3	3	3	3	3	0	n/a	n/a	3	3	3	0	0	0	27/48	56
Rautamo et al. [48]	3	3	3	3	3	3	3	3	n/a	n/a	3	3	3	3	0	3	39/48	81
Liu et al. [49]	0	3	3	0	2	3	1	3	3	3	n/a	3	1	n/a	0	3	28/48	58
Goyanes et al. [50]	1	3	3	0	1	3	1	0	1	3	n/a	3	1	n/a	0	2	23/48	48
Zheng et al. [51]	0	0	1	0	0	2	1	0	0	0	n/a	0	1	n/a	0	0	5/48	10
**Mean**	**1.2**	**2.5**	**2.7**	**1.2**	**1.8**	**2.8**	**2.0**	**1.3**	**1.0**	**2.3**	**3.0**	**2.5**	**2.0**	**1.5**	**0.5**	**1.7**		**55.0**

* QATSDD Criteria: (1) Explicit theoretical framework. (2) Statement of aims/objectives in main report. (3) Clear description of research setting. (4) Evidence of sample size considered in terms of analysis. (5) Representative sample of target group of a reasonable size. (6) Description of procedure for data collection. (7) Rationale for choice of data collection tool(s). (8) Detailed recruitment data. (9) Statistical assessment of reliability and validity of measurement tool(s) (Quantitative only). (10) Fit between research question and method of data collection (Quantitative only). (11) Fit between research question and format and content of data collection tool, e.g., interview schedule (Qualitative only). (12) Fit between research question and method of analysis. (13) Good justification for analytic method selected. (14) Assessment of reliability of analytical process (Qualitative only). (15) Evidence of user involvement in design. (16) Strengths and limitations critically discussed. Each QATSDD criteria is rated on a scale of 0–3 where: 0 = not at all achieved; 1 = very slightly achieved; 2 = moderately achieved; 3 = completely achieved.

## Appendix A. Search Strategy

Embase

Search conducted on 30 November 2023. Adapted and modified from [42].

**Search Number**	**Search Terms Used**	**Number of Results**
#1	exp ‘Printing, Three-Dimensional’/ or ‘Stereolithography’:tw.mp. or ‘Stereolithogr*’:tw.mp. or ((“3 D:tw” or ‘3D’:tw or ‘3 Dimensional’:tw or ‘3Dimensional’:tw or ‘Three Dimensional’:tw or ‘ThreeDimensional’:tw) and (‘Printing’:tw or ‘Print’:tw or ‘Print*’:tw)).mp. or ‘3D Manufacturing’:tw.mp. or ‘Three Dimensional Manufacturing’:tw.mp. or ‘3D Manufactur*’:tw.mp. or ‘Three Dimensional Manufactur*’:tw.mp. or ‘3D Produced’:tw.mp. or ‘3D Produc*’:tw.mp. or ‘Three Dimensional Produc*’:tw.mp. or ‘Printlets’:tw.mp. or ‘additive manufacturing’:tw.mp. or ‘additive manufactur*’:tw.mp. or ‘additive production’:tw.mp. or ‘additive produc*’:tw.mp. or ‘additive printing’:tw.mp. or ‘additive print*’:tw.mp. or ‘rapid prototyping’:tw.mp. or ‘rapid prototyp*’:tw.mp. or ‘bioprinting’:tw.mp. or ‘bioprint*’:tw.mp. or ‘Binder deposit*’:tw.mp. or ‘Binder deposition’:tw.mp. or ‘Binder jet*’:tw.mp. or ‘Binder jetting’:tw.mp. or ‘Direct ink writ*’:tw.mp. or ‘Direct ink writing’:tw.mp. or ‘Direct powder extru*’:tw.mp. or ‘Direct powder extrusion’:tw.mp. or ‘Drop on demand’:tw.mp. or ‘Drop on demand*’:tw.mp. or ‘Drop on drop’:tw.mp. or ‘Drop on drop*’:tw.mp. or ‘Drop on solid’:tw.mp. or ‘Drop on solid*’:tw.mp. or ‘Fused deposition model*’:tw.mp. or ‘Fused deposition modelling ‘:tw.mp. or ‘Fused filament fabric*’:tw.mp. or ‘Fused filament fabrication ‘:tw.mp. or ‘Ink jet*’:tw.mp. or ‘Ink jetting’:tw.mp. or ‘Inkjet print*’:tw.mp. or ‘Inkjet printing’:tw.mp. or ‘Micro extru*’:tw.mp. or ‘Micro extrusion’:tw.mp. or ‘On demand manufact*’:tw.mp. or ‘On demand manufacturing’:tw.mp. or ‘Pressure assisted microsyring*’:tw.mp. or ‘Pressure assisted microsyringe ‘:tw.mp. or ‘Rapid manufactur*’:tw.mp. or ‘Rapid manufacturing’:tw.mp. or ‘Selective laser sinter*’:tw.mp. or ‘Selective laser sintering ‘:tw.mp. or ‘Semi solid extru*’:tw.mp. or ‘Semi solid extrusion ‘:tw.mp. or ‘Vat photopolymer*’:tw.mp. or ‘Vat photopolymerization’:tw.mp. [mp=title, abstract, heading word, drug trade name, original title, device manufacturer, drug manufacturer, device trade name, keyword heading word, floating subheading word, candidate term word]	29,668
#2	(‘Tablets’:exp or ‘Dosage Forms’:exp or ‘Pharmaceutical Preparations’:exp or ‘Drug Therapy’:exp or ‘Pharmacology’:exp or ‘Pharmacokinetics’:exp or ‘Technology, Pharmaceutical’:exp or ‘Precision Medicine’:exp or ‘Personalized Medicine’:exp or ‘Individualized Medicine’:exp or ‘Extemporaneous preparations’:exp or ‘Extemporaneous preparation’:exp or (‘Tablets’ or ‘Dosage Forms’ or ‘Pharmaceutical Preparations’ or ‘Drug Therapy’ or ‘Pharmacology’ or ‘Pharmacokinetics’ or ‘Technology, Pharmaceutical’ or ‘chemotherapy’ or ‘dosage forms’ or ‘drug’ or ‘drug therapy’ or ‘drugs’ or ‘medication’ or ‘medications’ or ‘pharmaceutical’ or ‘pharmacokinetics’ or ‘pharmacology’ or ‘tablets’ or ‘chemotherap*’ or ‘dosage form’ or ‘medicat*’ or ‘pharmac*’ or ‘pharmacokinetic*’ or ‘pharmacol*’ or ‘formulation’ or ‘formulations’ or ‘pill’ or ‘pills’ or ‘Printlets’ or ‘Precision Medicine’ or ‘Personalized Medicine’ or ‘Personalised Medicine’ or ‘Individualized Medicine’ or ‘Individualised Medicine’ or ‘P Health’)).mp. [mp=title, abstract, heading word, drug trade name, original title, device manufacturer, drug manufacturer, device trade name, keyword heading word, floating subheading word, candidate term word]	1,444,235
#3	exp ‘Child’/ or ‘child’:tw.mp. or ‘children’:tw.mp. or exp ‘Infant’/ or ‘infant’:tw.mp. or ‘infants’:tw.mp. or ‘newborn’:tw.mp. or ‘newborns’:tw.mp. or ‘new-born’:tw.mp. or ‘newborns’:tw.mp. or ‘neonate’:tw.mp. or ‘neonates’:tw.mp. or ‘neonatal’:tw.mp. or ‘neo-nate’:tw.mp. or ‘neo-nates’:tw.mp. or ‘neo-natal’:tw.mp. or ‘neonatology’:tw.mp. or ‘NICU’:ti.mp. or ‘premature’:tw.mp. or ‘prematures’:tw.mp. or ‘pre-mature’:tw.mp. or ‘pre-matures’:tw.mp. or ‘preterm’:tw.mp. or ‘pre-term’:tw.mp. or ‘postnatal’:tw.mp. or ‘post-natal’:tw.mp. or ‘baby’:tw.mp. or ‘babies’:tw.mp. or ‘suckling’:tw.mp. or ‘sucklings’:tw.mp. or ‘toddler’:tw.mp. or ‘toddlers’:tw.mp. or ‘childhood’:tw.mp. or ‘schoolchild’:tw.mp. or ‘schoolchildren’:tw.mp. or ‘childcare’:tw.mp. or ‘child-care’:tw.mp. or ‘young’:ti.mp. or ‘youngster’:tw.mp. or ‘youngsters’:tw.mp. or ‘preschool’:tw.mp. or ‘pre-school’:tw.mp. or ‘kid’:tw.mp. or ‘kids’:tw.mp. or ‘boy’:tw.mp. or ‘boys’:tw.mp. or ‘girl’:tw.mp. or ‘girls’:tw.mp. or exp ‘Adolescent’/ or ‘adolescent’:tw.mp. or ‘adolescents’:tw.mp. or ‘adolescence’:tw.mp. or ‘pre-adolescent’:tw.mp. or ‘pre-adolescents’:tw.mp. or ‘pre-adolescence’:tw.mp. or ‘schoolage’:tw.mp. or ‘schoolboy’:tw.mp. or ‘schoolboys’:tw.mp. or ‘schoolgirl’:tw.mp. or ‘schoolgirls’:tw.mp. or ‘pre-puber’:tw.mp. or ‘pre-puberty’:tw.mp. or ‘prepuber’:tw.mp. or ‘prepubers’:tw.mp. or ‘prepuberty’:tw.mp. or ‘puber’:tw.mp. or ‘puberty’:tw.mp. or ‘puberal’:tw.mp. or ‘teenager’:tw.mp. or ‘teenagers’:tw.mp. or ‘teens’:tw.mp. or ‘youth’:tw.mp. or ‘youths’:tw.mp. or ‘underaged’:tw.mp. or ‘under-aged’:tw.mp. or exp ‘Pediatrics’/ or ‘Pediatric’:tw.mp. or ‘Pediatrics’:tw.mp. or ‘Paediatric’:tw.mp. or ‘Paediatrics’:tw.mp. or ‘PICU’:ti.mp. or (‘child’:ab,ti not child’:au).mp. or children*:ab,ti.mp. or schoolchild*:ab,ti.mp. or ‘infant’:ab,ti.mp. or ‘infants’:ab,ti.mp. or adolesc*:ab,ti.mp. or pediat*:ab,ti.mp. or paediat*:ab,ti.mp. or neonat*:ab,ti.mp. or toddler*:ab,ti.mp. or ‘teen’:ab,ti.mp. or ‘teens’:ab,ti.mp. or teenager*:ab,ti.mp. or preteen*:ab,ti.mp. or newborn*:ab,ti.mp. or postneonat*:ab,ti.mp. or postnatal*:ab,ti.mp. or ‘puberty’:ab,ti.mp. or preschool*:ab,ti.mp. or suckling*:ab,ti.mp. or ‘juvenile’:ab,ti.mp. or ‘new born’:ab,ti.mp. or ‘new borns’:ab,ti.mp. or new-born*:ab,ti.mp. or neo-nat*:ab,ti.mp. or neonat*:ab,ti.mp. or perinat*:ab,ti.mp. or underag*:ab,ti.mp. or ‘under age’:ab,ti.mp. or ‘under aged’:ab,ti.mp. or youth*:ab,ti.mp. or kinder*:ab,ti.mp. or pubescen*:ab,ti.mp. or prepubescen*:ab,ti.mp. or ‘prepuberty’:ab,ti.mp. or ‘school age’:ab,ti.mp. or ‘schoolage’:ab,ti.mp. or ‘school ages’:ab,ti.mp. or schoolage*:ab,ti.mp. or ‘one year old’:ab,ti.mp. or ‘two year old’:ab,ti.mp. or ‘three year old’:ab,ti.mp. or ‘four year old’:ab,ti.mp. or ‘five year old’:ab,ti.mp. or ‘six year old’:ab,ti.mp. or ‘seven year old’:ab,ti.mp. or ‘eight year old’:ab,ti.mp. or ‘nine year old’:ab,ti.mp. or ‘ten year old’:ab,ti.mp. or ‘eleven year old’:ab,ti.mp. or ‘twelve year old’:ab,ti.mp. or ‘thirteen year old’:ab,ti.mp. or ‘fourteen year old’:ab,ti.mp. or ‘fifteen year old’:ab,ti.mp. or ‘sixteen year old’:ab,ti.mp. or ‘seventeen year old’:ab,ti.mp. or ‘eighteen year old’:ab,ti.mp. or ‘1 year old’:ab,ti.mp. or ‘2 year old’:ab,ti.mp. or ‘3 year old’:ab,ti.mp. or ‘4 year old’:ab,ti.mp. or ‘5 year old’:ab,ti.mp. or ‘6 year old’:ab,ti.mp. or ‘7 year old’:ab,ti.mp. or ‘8 year old’:ab,ti.mp. or ‘9 year old’:ab,ti.mp. or ‘10 year old’:ab,ti.mp. or ‘11 year old’:ab,ti.mp. or ‘12 year old’:ab,ti.mp. or ‘13 year old’:ab,ti.mp. or ‘14 year old’:ab,ti.mp. or ‘15 year old.mp. [mp=title, abstract, heading word, drug trade name, original title, device manufacturer, drug manufacturer, device trade name, keyword heading word, floating subheading word, candidate term word]	4,048,728
#4	exp ‘Neoplasms’/ or ‘Cancer’:tw.mp. or ‘haematolog*’:tw.mp. or ‘hematolog*’:tw.mp. or ‘oncolog*’:tw.mp. or ‘Tumour’:tw.mp. or ‘Tumor’:tw.mp. or ‘Tumo*’:tw.mp. [mp=title, abstract, heading word, drug trade name, original title, device manufacturer, drug manufacturer, device trade name, keyword heading word, floating subheading word, candidate term word]	5,607,776

## Appendix B. Studies Excluded Following Full Text Review

1. Boudriau S, Hanzel C, et al. Randomized Comparative Bioavailability of a Novel Three-Dimensional Printed Fast-Melt Formulation of Levetiracetam Following the Administration of a Single 1000-mg Dose to Healthy Human Volunteers Under Fasting and Fed Conditions. Drugs R D. **2016** Jun;16(2):229-38. doi: 10.1007/s40268-016-0132-1. PMID: 27028750; PMCID: PMC4875927.
  *Reason for exclusion: Not in children*
2. Karavasili C, Zgouro P, et al. Cereal-Based 3D Printed Dosage Forms for Drug Administration During Breakfast in Pediatric Patients within a Hospital Setting. J Pharm Sci. **2022** Sep;111(9):2562-2570. doi: 10.1016/j.xphs.2022.04.013. Epub **2022** Apr 22. PMID: 35469835.
  *Reason for exclusion: Not in vivo*
3. van Kampen EEM, Willemsteijn L, et al. 3D printing of drugs: expanding the options for child-tailored pharmacotherapy. Arch Dis Child. **2021** Jul 14:archdischild-2021-321629. doi: 10.1136/archdischild-2021-321629. Epub ahead of print. PMID: 34261670.
  *Reason for exclusion: Not primary data*
4. Tabriz AG, Hui HW, et al. 3D Printed Flavor-Rich Chewable Pediatric Tablets Fabricated Using Microextrusion for Point of Care Applications. Mol Pharm. **2023** Jun 5;20(6):2919-2926. doi: 10.1021/acs.molpharmaceut.2c01061. Epub 2023 Apr 6. PMID: 37022302.
  *Reason for exclusion:* Palatability assessment, but only in healthy adult volunteers not in children.
5. Tabriz AG, Fullbrook DHG, et al. Personalised Tasted Masked Chewable 3D Printed Fruit-Chews for Paediatric Patients. Pharmaceutics. **2021** Aug 20;13(8):1301. doi: 10.3390/pharmaceutics13081301. PMID: 34452262; PMCID: PMC8400795.
  *Reason for exclusion:* Palatability assessment, but only in healthy adult volunteers not in children.
6. Blazhenkova O, Dogerlioglu-Demir K. The shape of the pill: Perceived effects, evoked bodily sensations and emotions. PLoS One. **2020** Sep 8;15(9):e0238378. doi: 10.1371/journal.pone.0238378. PMID: 32898184; PMCID: PMC7478620.
  *Reason for exclusion:* Palatability assessment, but in healthy adult volunteers not in children.
